# A Method for the Generation of Ectromelia Virus (ECTV) Recombinants: *In Vivo* Analysis of ECTV vCD30 Deletion Mutants

**DOI:** 10.1371/journal.pone.0005175

**Published:** 2009-04-13

**Authors:** Ali Alejo, Margarida Saraiva, Maria Begoña Ruiz-Argüello, Abel Viejo-Borbolla, Mar Fernández de Marco, Francisco Javier Salguero, Antonio Alcami

**Affiliations:** 1 Centro de Investigación en Sanidad Animal, Instituto Nacional de Investigación y Tecnología Agraria y Alimentaria, Valdeolmos, Madrid, Spain; 2 Department of Medicine, University of Cambridge, Addenbrooke's Hospital, Cambridge, United Kingdom; 3 Centro de Biología Molecular Severo Ochoa (Consejo Superior de Investigaciones Científicas and Universidad Autónoma de Madrid), Cantoblanco, Madrid, Spain; Karolinska Institutet, Sweden

## Abstract

**Background:**

Ectromelia virus (ECTV) is the causative agent of mousepox, a lethal disease of mice with similarities to human smallpox. Mousepox progression involves replication at the initial site of infection, usually the skin, followed by a rapid spread to the secondary replicative organs, spleen and liver, and finally a dissemination to the skin, where the typical rash associated with this and other orthopoxviral induced diseases appears. Case fatality rate is genetically determined and reaches up to 100% in susceptible mice strains. Like other poxviruses, ECTV encodes a number of proteins with immunomodulatory potential, whose role in mousepox progression remains largely undescribed. Amongst these is a secreted homologue of the cellular tumour necrosis factor receptor superfamily member CD30 which has been proposed to modulate a Th1 immune response *in vivo*.

**Methodology/Principal Findings:**

To evaluate the contribution of viral CD30 (vCD30) to virus pathogenesis in the infected host, we have adapted a novel transient dominant method for the selection of recombinant ECTVs. Using this method, we have generated an ECTV vCD30 deletion mutant, its corresponding revertant control virus as well as a virus encoding the extracellular domain of murine CD30. These viruses contain no exogenous marker DNA sequences in their genomes, as opposed to other ECTVs reported up to date.

**Conclusions/Significance:**

We show that the vCD30 is expressed as a secreted disulfide linked trimer and that the absence of vCD30 does not impair mousepox induced fatality *in vivo*. Replacement of vCD30 by a secreted version of mouse CD30 caused limited attenuation of ECTV. The recombinant viruses generated may be of use in the study of the role of the cellular CD30-CD30L interaction in the development of the immune response. The method developed might be useful for the construction of ECTV mutants for the study of additional genes.

## Introduction

Poxviruses are a family of large, complex shaped enveloped dsDNA viruses of cytoplasmic replication [1]. They are subdivided into two subfamilies, depending on whether their host are invertebrates (*Entomopoxvirinae*) or vertebrates (*Chordopoxvirinae*). The *Chordopoxvirinae* are further classified into eight genera according to their antigenic properties and genome sequences. Among the orthopoxviruses are several species capable of infecting humans such as variola virus (VARV), the causative agent of smallpox, vaccinia virus (VACV), the virus which was used to erradicate smallpox from the human population by mass vaccination, or monkeypox virus (MPXV), a poxvirus causing a disease with similarities to smallpox but reduced case fatality rates which has raised some concern about potential poxviral zoonoses [2]. Ectromelia virus (ECTV) is a mouse specific pathogen that belongs to the orthopoxvirus genus and causes mousepox, a severe disease with similarities to smallpox, in susceptible mouse strains. ECTV was used as a model for VARV infections and served to establish the course of acute, systemic viral infections [3], [4]. ECTV usually enters its host through abrasions in the skin, where it replicates extensively and migrates to the draining lymph node. From this initial replication site, the virus further spreads to the central target organs, spleen and liver. A second hematogenous spread from these sites to the skin produces the rash and pocks which are characteristic of mousepox and other poxviral diseases. Susceptibility to mousepox is genetically controlled and has been mapped to the resistance to mousepox (rmp) loci which include genes near the natural killer gene complex [5]. In susceptible strains, such as BALB/c, the infected mice usually die from acute liver necrosis at around day 8 to 10 postinfection, before the secondary viremia takes place. Resistant strains, such as C57BL6, however, develop no apparent signs of illness and viral replication is effectively controlled by the host. Much effort has been devoted recently to study the mechanisms of immune response against ECTV. It has been shown that an early control of infection by natural killer (NK) cells in resistant mice is essential to avoid mousepox lethality [6]. The transition to an effective cytotoxic T lymphocyte (CTL) and antibody response as well as a polarized Th1 response have been demonstrated to be essential for survival of the infected host and clearance of the virus [7]–[9].

Due to their large coding capacity, poxviruses have developed a set of strategies to modulate their interaction with the host. Indeed, around one third of the approximately 200 genes encoded by a prototypical poxvirus are predicted to be directly involved in the modulation of the host immune response. Out of these, several were found to correspond to secreted proteins which can act as either cytokine receptor homologues (viroceptors) or as cytokine mimics (virokines). Examples of viroceptors include the VACV secreted interleukin 1β (IL-1β) binding protein B15 [10], [11] and the interferon (IFN) type I binding protein B18 [12], [13]. Virokines such as the secreted VACV A39 smaphorin, which induces cytokine production from monocytes [14] or the secreted IL-10 homologues found in orf [15] and Yaba-like disease (YLDV) viruses [16]. Immune evasion mechanisms and their role on the pathogenesis of poxviruses are extensively reviewed in [17]–[19].

One interesting example of viroceptors is the family of viral tumor necrosis factor receptors (TNFRs) (reviewed in [20]([21]TNF is an important proinflammatory cytokine with an essential role in protection against invading pathogens. Not surprisingly, most pathogens have developed means to block or take advantage of TNF and TNF signalling in their infected hosts (reviewed by [22]). Poxviruses have developed a family of secreted TNFRs which are thought to act *in vivo* by binding to TNF and blocking its activity. Indeed, most poxviral species have been predicted to encode at least one active member of this family, underscoring the importance of the TNF-TNFR axis in the control of viral infections. Viral TNFRs belong to two different families. The YLDV 2L protein-like proteins bind to TNF but share no sequence similarity to cellular TNFRs or to other cellular genes of known or unknown function [23], [24]. The cytokine response modifier B (CrmB), CrmC, CrmD and CrmE proteins, which are differentially expressed by poxviral species all contain a variable number of the cysteine rich domains (CRDs) which define the superfamily of cellular TNFRs. All of them have been described to bind and inhibit TNF and/or lymphotoxin *in vitro* [25]–[29]. A fifth member of this viral protein family containing two CRDs was described as a viral CD30 homologue (vCD30) [30], [31]. This protein is most similar to the murine CD30 protein and was found to interact not with TNF but with CD30 ligand (CD30L, CD153), a distinct member of the TNF superfamily which is the only described ligand for cellular CD30.

ECTV vCD30 was described as a 12-kDa, secreted late viral protein. It binds murine CD30L with an affinity of 0.66 nM and is able to block the binding of CD30L to its cell surface receptors. Moreover, vCD30 was found to be able to induce reverse signalling through the CD30L expressed on the surface of freshly isolated human neutrophils [31]. This is a unique property among viral TNFR superfamily members, which are thought to act as decoy receptors, blocking the interaction of a soluble cytokine with its cognate receptor. Soluble CD30L, however, has not been described so far and therefore vCD30 must be acting at the cell surface either interfering with the interaction of CD30 with CD30L and or inducing signalling through CD30L. Finally, vCD30 was found to inhibit the activation of IFN-γ production by splencocytes in a mixed lymphocyte reaction and to block a Th1 but not a Th2-like inflammatory response *in vivo* [31]. Therefore, vCD30 was proposed to act as an important determinant of mousepox pathogenesis *in vivo*.

To test this hypothesis, we wished to construct a recombinant ECTV lacking the vCD30 gene. The generation of recombinant poxviruses relies on homologous recombination within the cell between viral sequences present in the replicating virus genomes and viral DNA sequences present in a transfected plasmid. Construction of deletion mutants in poxviruses can be achieved by disruption or replacement of the open reading frame of interest with a selectable marker. In spite of being an easy and fast to achieve method, the introduction of a very active new transcriptional unit may affect expression levels of neighbouring genes [32] and the production of a foreign marker may affect virus replication *in vivo*. Alternatively, deletion can be achieved by transient-dominant selection of the recombinant viruses and, although technically more complex, the results obtained are more trustable since the deletion of the gene is less likely to cause a phenotype not mediated by the gene of interest. In addition, this method for the deletion of a gene facilitates the production of a revertant control virus in which a full-length gene is reinserted into the same locus [33].

As the frequency of the recombinant viruses formed upon homologous recombination is low, recombinant viruses have to be distinguished from the parental viruses, which constitute the vast majority of the virus progeny. With this purpose, a variety of genetic markers have been used in the past, including colour selection, by using β-galactosidase, β-glucuronidase or EGFP, and specific drug selection, such as neomycin or mycophenolic acid. Although the presence of a coloured marker is easier to recognise, the presence of a selectable marker that confers resistance to a drug facilitates selection of the recombinant viruses. A combination of both criteria was developed to obtain VACV recombinants by using the EGFP and the puromycin acetyltransferase (*pac*) gene from *Streptomyces alboniger*, which confers resistance to the inhibitor of protein synthesis puromycin [34].

In this study, we have adapted this method to efficiently generate ECTV deletion mutants lacking any foreign sequence in their genomes. Additionally, the method allows the consecutiev modification of recombinant viruses without the need for additional selectable markers at each recombination step. This method has been used to study the role of vCD30 during mousepox progression.

## Results and Discussion

### A transient dominant selection method for the generation of recombinant ECTV

**Figure 1:**
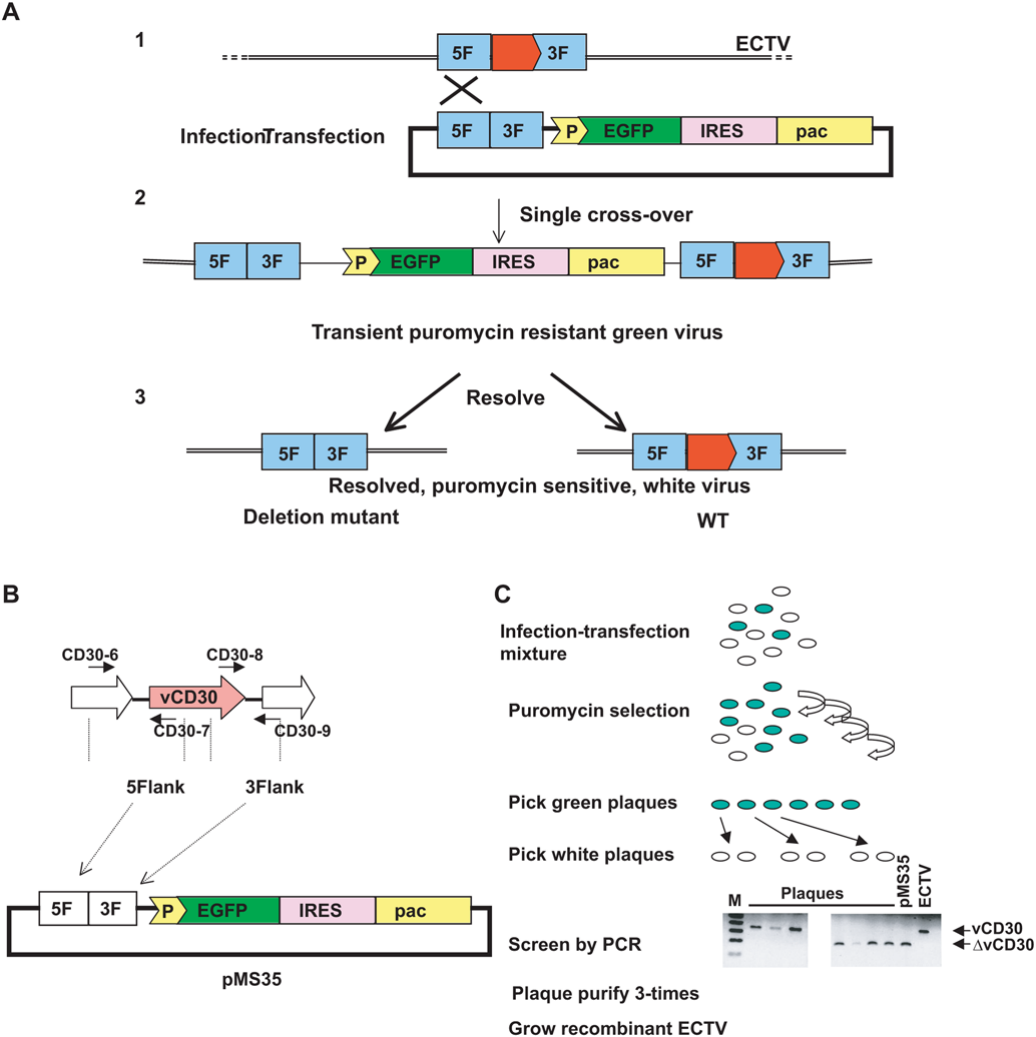
Transient dominant selection for the generation of ECTV recombinant viruses. A. Schematic representation of the recombination events between the viral genome and the plasmid as well as the phenotypes of intermediate and final viruses obtained during the procedure. B. Representation of the plasmid pMS35 used for the generation of the ECTVΔCD30 virus. The *vCD30* locus and the primers used for amplification of the flanking regions are indicated. C. Flowchart of the main steps in the generation of the ECTVΔCD30 virus.

The study of the pathogenesis of orthopoxviruses lacking particular genes involved in the evasion of the immune response provides important information not only on the role of these genes in the virus context but also on the host antiviral defence mechanisms [35]. We have adapted the method described by Sanchez-Puig et al. [34] for the generation of recombinant ECTVs. The principle of the method (Figure 1A) consists on the simultaneous infection of cells with ECTV and transfection with a recombinant plasmid containing approximately 500 bp of the 5′ and 3′ flanking regions of the gene to be deleted and a downstream expression cassette that allows for selection (Figure 1B). Homologous recombination between viral DNA sequences present in the recombinant plasmid and genomic ECTV DNA produces recombinant viruses formed by a single cross-over event that inserts the complete plasmid into the ECTV genome. The intermediate viral species transiently expresses the selectable markers, EGFP, which can be visualized under the fluorescence microscope and pac, which confers resistance to puromycin. This intermediate virus can be isolated by allowing the virus to replicate during several passages and to be amplified in the presence of the antibiotic, which causes cells to detach and does not allow plaque assays. A subsequent plaque assay on cell monolayers in the absence of puromycin selection enables picking of plaques which appear green under UV-light examination (Figure 1C). In a second step, perfomed on cell monolayers in the absence of selection, another recombination event occurs, resulting in the resolution of the intermediate virus and leading to the formation of either a virus lacking the targeted gene or to wild-type virus. Providing that the length of the flanking regions cloned into the recombinant plasmid is similar, the proportion of mutant and wild-type viruses should be similar. At this stage, it is possible to isolate viral plaques that have lost the selectable marker and test them in order to identify a deletion mutant virus and to distinguish it from wild-type viruses. Finally, the desired recombinant virus is purified by successive plaque purification. The production and selection of a revertant virus is achieved exactly in the same way, but in this case the cells are infected with the deletion mutant, instead of the wild-type virus, and transfected with a plasmid containing a full-length wild-type copy of the gene and its flanking regions. In the first step, an intermediate virus, harbouring the fully integrated recombinant plasmid and both the wild type and deleted versions of the gene, is selected by serial passage in the presence of puromycin. In the second step, virus plaques expressing a wild type gene are isolated by plaque assay in the absence of puromycin.

The initial vector described by Sanchez-Puig et al. [34] was optimised for the use with ECTV by replacement of the synthetic early/late promoter that was driving the expression of EGFP for the stronger synthetic late promoter [36], which appears to work better in ECTV infection (N. Bryant and A. Alcami, unpublished results). Additionally, in order to achieve good levels of pac expression, we cloned the IRES of the encephalomyocarditis virus between the *EGFP* and *pac* genes which allowed the bicistronic mRNA transcript to be translated from the 5′ end and from the IRES sequence, so that the *pac* gene was also under the control of the strong synthetic late promoter. This selectable *cassette* was transferred to pUC118, so that the flanking regions of the genes of interest could be cloned upstream of it. This final universal vector for the construction of ECTV recombinant viruses was named pMS30. Further optimisation steps of the method were required to generate recombinant ECTVs and included adjusting the concentration and time of addition of puromycin so that the growth of the recombinant viruses over the wild-type background was favoured (data not shown). The necessary number of puromycin selection steps was estimated at four. Titration of the viruses present after each puromycin selection cycle showed that the percentage of green virus increased until the fourth step and was constant after that (data not shown). The percentage of green ECTV never reached 100%, most likely due to the continuous resolution of the intermediate puromycin-resistant virus caused by the presence of direct repeats and the occurance of a second recombination event rendering both deletion mutant and wild-type viruses white (data not shown).

Circular plasmids containing the full-length VACV genome that could be stably propagated in *E. coli* and converted to infectious virus in mammalian cells were more recently developed [37]. The authors proposed that this would make it possible to modify or delete VACV genes or add foreign DNA in a bacterial system, eliminating the need for the plaque purification procedures employed up to date, providing a more efficient method for high throughput analyses. This procedure was recently adapted to study the role of five proteins with potential immunomodulatory properties of the attenuated strain Modified Vaccinia virus Ankara (MVA) [38], which is widely used as a safe recombinant vaccine vector. Although the method allowed the rapid generation and analysis of deletion mutants, the viruses generated still contained foreign marker sequences. Thus, this approach may be more useful for screening analyses or generation of large recombinant virus libraries in different poxviruses, including ECTV.

### Generation of recombinant ECTV lacking the *vCD30* gene

**Figure 2:**
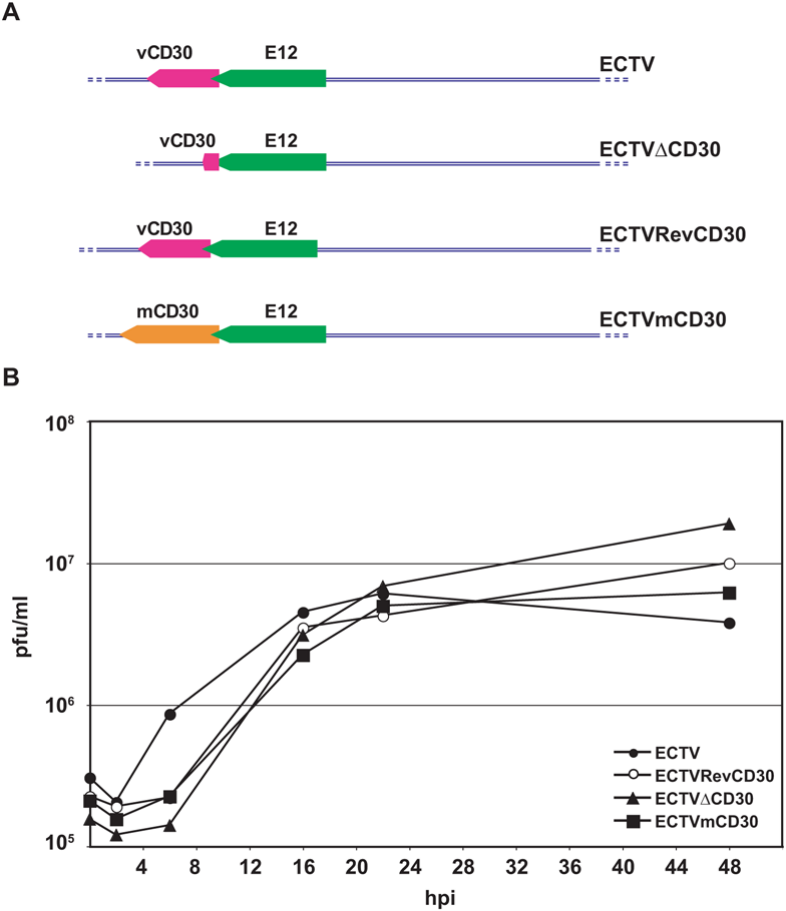
ECTV vCD30 is not essential for virus growth in cell culture. A. Representation of the genomic structure of the different ECTV generated showing the vCD30 and the adjacent E12 loci. B. Single-step growth curves of the indicated viruses on BSC-1 cells. Shown are means of triplicate samples for each time point.

The method described was used for the construction of recombinant ECTVs which are schematically depicted in Figure 2A. In the first place, an ECTV deletion mutant in the vCD30 gene (ECTVΔCD30) was obtained. To control for unwanted mutations elsewhere in the genome of ECTVΔCD30 and for specificity of any observed phenotype, this deletion mutant was used to generate a revertant virus (ECTVRevCD30) in which a copy of the *vCD30* gene is reinserted into its locus. Finally, to compare the properties of the vCD30 with those of the cellular secreted form of CD30, we introduced a copy of the region encoding the secreted domain of murine CD30 under the control of the ECTV vCD30 promoter into the orignal *vCD30* locus, generating ECTVmCD30.

As shown in Figure 2B, all recombinant viruses grew with similar kinetics to the parental ECTV on BSC-1 cell monolayers, showing that lack of vCD30 or expression of murine CD30 does not affect viral replication in cell culture.

Only a few recombinant ECTVs have been generated and characterized so far. Deletion of the ECTV homologue of the VACV host range gene K1L was achieved by insertion of the *E. coli* guanine phosphoribosyltransferase (*Eco-gpt*) gene and selection of the recombinant virus in the presence of mycophenolic acid [39]. Infections of mice with the K1L-lacking ECTV yielded a pathogenesis pattern indistinguishable from wild-type, suggesting that the ECTV homologue of VACV K1L is not important for ECTV replication and spread *in vivo*. An ECTV thymidine kinase (TK) minus recombinant obtained by insertion of the β-galactosidase gene into the viral TK gene showed decreased virulence possibly due to a decreased replicative capacity in target cells *in vivo* [40]. An ECTV virus lacking the p28 protein, which has been later found to act as an E3 ubiquitin ligase [41] was obtained by insertion of the *Eco-gpt* gene [42] and found to be attenuated *in vivo*. This attenuation was attributed to a failure of the virus to replicate in macrophage lineage cells, impairing viral spread from the skin to its target organs [43]. The same method was used to obtain an ECTV mutant lacking the IL-18 binding protein, p13 [44]. Although this virus showed an increased NK cell activity, only a modest attenuation in terms of viral titers was described. The main criticism to these studies is the absence of a revertant virus, in which the wild type gene is reinserted into the viral genome, to control for inadvertent mutations present elsewhere in the deletion mutant. All the referred recombinant ECTVs were generated by insertion of a selectable marker into the gene of interest and therefore the resulting phenotype may not necessarily be the only consequence of the absence of the targeted gene. The more recently described deletion mutant of the ECTV IFN-γ binding protein was obtained by a transient dominant selection method using *Eco-gpt* [45]. In this case, a revertant virus was generated. However, the deletion mutant still contained an exogenous *LacZ* in its genome. For the deletion of the type I IFN binding protein encoded by EVM166, insertion of the EGFP gene marker was used [46]. Again, a revertant virus was generated, but lacked the EGFP present in the virus which it was compared to. Thus, we believe that the method presented here for the generation of recombinant ECTVs is of benefit for the study of the virus *in vivo*, as the viruses to be compared will differ only in the genes of interest, containing no exogenous sequences in their genomes. Additionally, viral genomes may be sequentially modified using the same approach, as selctable markers are not retained and can therefore be reused.

### The secreted vCD30 interacts specifically with CD30L and forms disulfide-mediated trimers

**Figure 3:**
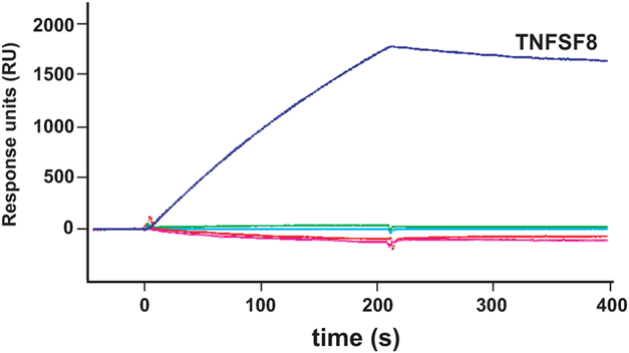
ECTV vCD30 ligand screening. Sensorgram showing binding of mTNFSF8 (CD30L) injected at 50 nM to purified vCD30 coupled to a CM5 sensor chip by SPR analysis. TNFSF10 (pink line), TNFSF11 (cyan line), TNFSF12 (red line) and TNFSF14 (green line) injected at 100 nM each did not show interaction.

To study the role of ECTV vCD30, we first expressed and purified a His-tagged vCD30 protein (vCD30His) using a baculovirus expression system. By non-reducing SDS-PAGE we observed that this recombinant protein formed disulfide-mediated trimers in solution (not shown). We next screened this protein for potential binding partners among the members of the TNF superfamily of murine origin. Previous reports had shown that the vCD30Fc fusion protein did not bind to human or murine TNFα (TNFSF2), Ltα (TNFSF1), LTβ (TNFSF3), LTαβ complexes or the ligands for CD40 (TNFSF5), 4-IBB (TNFSF9), OX40 (TNFSF4), Fas (TNFSF6) or CD27 (TNFSF7) in competition by scintillation proximity assay (FlashPlate) [30], [31]. We confirmed by surface plasmon resonance (SPR) with a BIAcore X biosensor this lack of interaction for the purified vCD30His protein and extended it to the murine RANK L (TNFSF 11), TWEAK (TNFSF 12), APRIL (TNFSF 13), LIGHT (TNFSF 14) proteins (Figure 3). Thus, we extended previous data to a larger set of 15 TNFSF members and concluded that CD30L (TNFSF 8) is the sole binding partner of vCD30 (Figure 3 and not shown).

**Figure 4:**
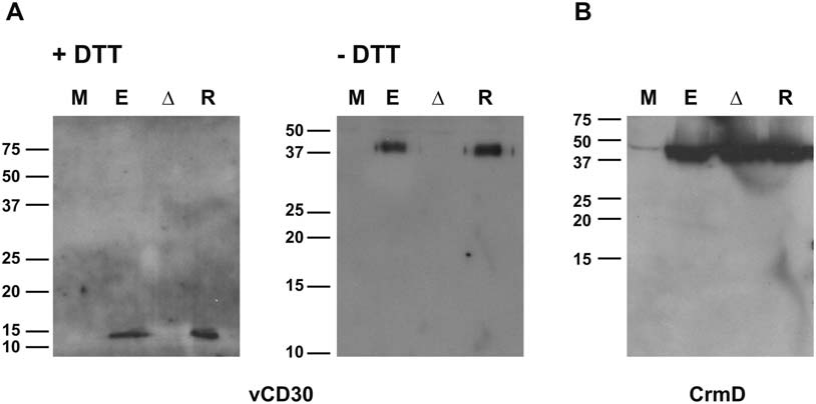
ECTV vCD30 forms disulfide linked trimers. Western blot analysis using anti vCD30 (A) or anti CrmD (B) antibodies of supernatants from mock-infected BSC-1 cells (M) or cells infected with ECTV (E), ECTVΔCD30 (Δ) or ECTVRevCD30 (R).

We next tested whether the endogenous vCD30 protein was secreted as a trimer during ECTV infection of cells. Western blot analyses of supernatants of BSC-1 cells infected with parental ECTV or ECTVRevCD30 and harvested at late times post-infection showed a band of approximately 12 kDa which was absent from samples from ECTVΔCD30 infected cells. This band migrated at approximately 38 kDa in the absence of a reducing agent, showing that the vCD30 synthesised during infection forms disulfide bridge-mediated multimers most likely corresponding to trimers (Figure 4A). As expected, the samples obtained from the ECTVRevCD30-infected cells showed similar expression levels of vCD30. A control western blot revealed similar amounts of the late secreted ECTV protein CrmD in the samples of all infected cells (Figure 4B). It has been shown that cellular TNFRs undergo trimerization upon or prior to ligand binding [47], [48], which is important for intracellular signalling. The vCD30 may have adapted this strategy to generate a protein with a stable conformation mediated by covalent bonds which may act as a more potent ligand for the cognate cellular receptor or for displacement of the cellular CD30-CD30L interaction. In this regard it has been shown that the related viral TNFR CrmD from ECTV is capable of forming disulfide-linked complexes which can bind their ligand [28]. The VARV secreted TNFR CrmB was also found to form disulfide mediated dimers [49]. Similarly, it has been found that the ECTV secreted IFN-γ binding protein forms tetramers which are critical for antagonizing IFN-γ activity [50].

### Role of vCD30 in mousepox

To study the contribution of vCD30 to ECTV virulence, we first infected resistant C57BL6 or susceptible BALB/c mice subcutaneously in the footpad with different doses of the mentioned wild type or recombinant viruses. In the case of C57BL6 mice, doses of 10^4^, 10^5^ and 10^6^ pfu per animal were tested and no differences were observed in weight loss or signs of illness over the 30 day observation period, with all mice resisting to infection but showing swelling of the inoculation site, indicative of active viral replication (not shown). Resistant mice are able to mount an efficient Th1 response in the presence of vCD30 and therefore were not expected to succumb to infection with the ECTVΔCD30 mutant. A similar set of experiments was perfomed using susceptible, BALB/c mice, which are characterised by developing a poor Th1 response to ECTV. As before, signs of illness, weight loss and footpad swelling were scored daily during a 35 day post-inoculation period. After subcutaneous infection of susceptible BALB/c mice with doses of 1, 10, 100 and 1000 pfu per animal, an approximate LD_50_ of 10 pfu and a mean time to death of 11 days post-infection were observed in all groups (not shown). This indicated that vCD30 is not required for mousepox induced lethality or disease severity. The vCD30 has been shown to be an effective inhibitor of Th1-like inflammatory responses *in vivo* [31] suggesting that it may be compromising an effective antiviral response during mousepox. Although it remains to be established whether the immune response in ECTVΔCD30 infected mice was quicker or more potent in terms of activation, these possible differences were not able to overcome infection. In this regard it would be interesting to determine the LD_50_ of the different viruses in mouse strains of intermediate susceptibility. Expression of the soluble murine CD30 did not have any effect in any of the situations examined.

**Figure 5:**
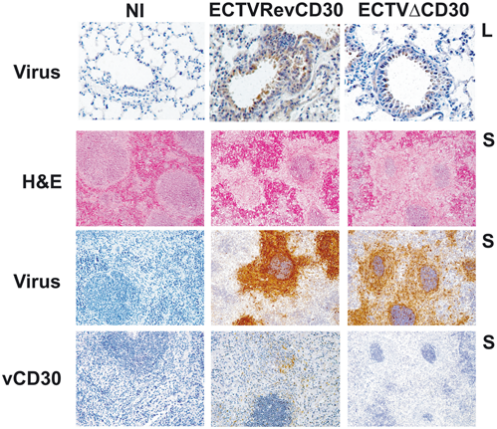
Absence of ECTV vCD30 does not affect viral spread or replication *in vivo*. Lung (top row, L) and spleen (S) samples from representative uninfected (NI) or intranasally infected BALB/c mice at day 7 post-infection were analysed by IHC for virus replication and vCD30 expression, as indicated. H&E analysis shows extensive necrosis of spleen tissue of infected animals.

**Figure 6:**
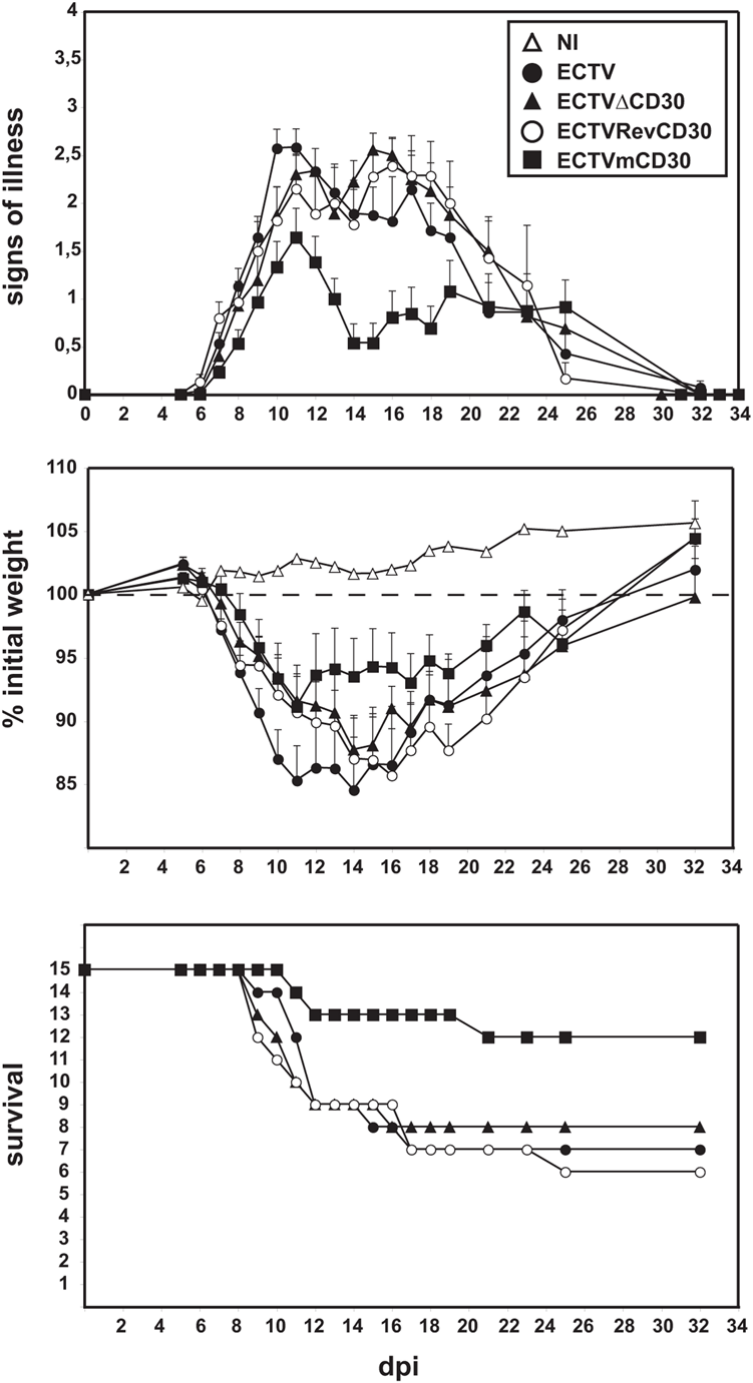
Expression of murine soluble CD30 attenuates mousepox development. Groups of 15 susceptible BALB/c mice were infected intranasally with 100 pfu each of the indicated viruses and scored daily for signs of illness and weight loss. Survival rates are shown on the bottom panel.

The natural infection route for ECTV is through abrasions in the skin and therefore subcutanous inoculation is possibly the most physiologically relevant model. However, oronasal transmission is also possible. Therefore, we next tested the intranasal inoculation route. The resistant C57BL6 strain shows an enhanced susceptibility to ECTV when infected intranasally. When C57BL6 mice were inoculated intranasally with high doses of parental ECTV virus (10^4^, 10^5^ and 10^6^ pfu per animal), all infected mice died after a 9 to 12 day period. No significant differences were observed among the animals infected with the different recombinant viruses (not shown). Intranasal infection of susceptible BALB/c mice with doses of 1, 10 or 100 pfu per animal showed no significant differences between ECTV or ECTVΔCD30-infected mice, with the LD_50_ at approximately 100 pfu and mean time to death of 12 days post-infection. As shown in Figure 5, ECTV replication in the bronchiolar epithelia was readily detected by day 7 post-infection in animals infected with either parental, ECTVΔCD30 or ECTVRevCD30 viruses. Concomitant spleen necrosis and replication in this target organ was also detected by immunohistochemistry (IHC). Expression of vCD30 *in vivo* was similarly confirmed by IHC (Figure 5). Infection with ECTV mCD30 appeared to result in a slightly attenuated disease at these low doses, and to confirm this difference a second experiment using groups of 15 susceptible mice was performed (Figure 6). Indeed, a modest reduction of mortality in the case of ECTV mCD30 infected mice as compared to the parental ECTV, ECTVΔCD30 or control ECTVRevCD30 infected mice was observed. The reason for this slight attenuation is currently unknown and may relate to differences in the specific activity of the vCD30 as compared to the mCD30.

Thus, we have shown that the ECTV vCD30 is not a major determinant of mousepox induced mortality in susceptible BALB/c mice and that deletion of the gene does not affect the resistant host's ability to overcome the viral infection. It remains to be established whether the quantity or the quality of the immune response against the invading virus is affected in the absence of the vCD30 protein and whether viral replication is compromised under these conditions. In this context it is important to mention that CD153 (CD30L) was shown to be important in the augmentation of a Th1 response capable of producing IFN-γ against *Mycobacterium bovis* bacillus Calmette-Guerin (BCG) infection in mice, with the number of bateria being significantly higher in the organs of CD153 knockout mice than in wild type mice [51]. Additionally, other roles for the CD30-CD153 pair with potential impact on mousepox development have been shown such as modulation of B-cell responses [52], [53] and memory T cell generation [54], suggesting other aspects of the immune response against ECTV that will have to be studied in the future to determine the mode of action of the vCD30.

Although it has been considered that secreted viral proteins with immunomodulatory potential may act as essential determinants of viral pathogenesis, few such proteins have been analysed in detail during ECTV infection. Thus, deletion of the ECTV IL-18 binding protein produced better NK cell activation and higher levels of type I cytokines during infection, but had no significant impact on viral replication *in vivo* [44]. In contrast, deletion of the type I IFN [46] or type II IFN soluble receptors [45] had an important impact on both immune response and host's survival, as exemplified by the difference in LD_50_ in susceptible mice in both cases. It will be of interest to establish the relative contribution of other secreted immunmodulators such as the TNF and chemokine receptor CrmD, the complement inhibitory protein or the chemokine inhibitory protein among others.

The vCD30 was originally described in cowpox virus and ECTV strains, although the sequencing of new poxviral genomes has found vCD30 orthologues which are predicted to be active in horsepox virus [55], a probable predecessor of VACV [56] and the W83, but not the W84 strain of deerpox virus [57]. In any case, the presence of vCD30 orthologues is restricted to a few poxvirus species, suggesting either a common evolutionary history or an unknown common host-related selective pressure for all of these viral species. We have generated a valuable tool for the determination of the role of the vCD30 during poxvirus-caused infection in their hosts. Additionally, we expect that the recombinant viruses that we have generated may be used to analyse the role of the cellular CD30-CD30L interaction during acute viral infections *in vivo*.

## Materials and Methods

### Cells and viruses

The reference ECTV strain used was ECTV Naval.Cam (complete genome sequence available at www.poxvirus.org). This virus was derived from the original ECTV Naval strain obtained from Dr. Mark Buller (Universiy of St. Louis, USA) by isolating virus from a spleen sample of an infected BALB/c mouse by three consecutive rounds of plaque purification on BSC-1 cell monolayers. ECTVs were grown in BSC-1 cells. For infection of mice, virus stocks were purified by centrifugation through a 36% sucrose cushions as described [58].

### Construction of recombinant ECTVs

The plasmid pMS30 was constructed for expression of EGFP under a synthetic late promoter followed by an IRES cassette for expression of the puromycin acetyltransferase gene from the same transcript. Details of the sequence and generation of the plasmid are available upon request. The 5′ flanking region of the *CD30* gene was amplified with oligonucleotides CD30-6 (5′-GCGGAATTCCTGATAGACAGTTGCCTATTAAAGTG) and CD30-7 (5′-GCGGGATCCGATGTACTCCTGATACCACAACAAAG), and the 3′ flanking region of CD30 was amplified with oligonucleotides CD30-8 (5′-GCGGGATCCGATGTATATACTAGGCAGGTTACG) and CD30-9 (5′-GCGCTGCAGCATCGATAAATTGACCAAGTTACAC). Both flanking regions were cloned sequentially into EcoRI- and PstI-digested pMS30 to give plasmid pMS35, which was used for the generation of ECTVΔCD30. Both flanking regions and the intervening *CD30* gene were PCR-amplified with oligonucleotides CD30-6 and CD30-9 to generate the plasmid pMS38 that was used for reinsertion of the *CD30* gene into the ECTVΔCD30 genome and construction of ECTVRevCD30. To construct ECTVmCD30, a new 3′flanking region for vCD30 which contained the putative promotor of the vCD30 gene but not its initiator ATG was amplified with oligonucleotides CD30-9 and CD30-11 (5′-GCGGGATCCGCTATTTAATACATCTAATATATG) into BamHI- and PstI-digested pMS35, giving plasmid pAH6. The sequence coding for the predicted extracellular domain of the murine CD30 (M1 to T281) was PCR-amplified with oligonucleotides MmCD30-7 (5′-CGCGGATCCAGCATGAGCGCCCTACTCACCGCAGC) (including a consensus Kozak sequence) and MmCD30-8 (5′- GCGGGATCCTATGTTCCCGTGGACAATGGAGAGGTG) (including a stop codon) and cloned into BamHI-digested pAH6, giving plasmid pAH10. The plasmid PBMGNeo mCd30 full length used as a template was kindly provided by Dr. E. Podack (University of Miami, USA). All plasmids were sequenced to confirm absence of unwanted mutations. To generate the recombinant ECTVs, BSC-1 cells were transfected with pMS35, pMS38 or pAH10 using Genejammer (Stratagene) following the manufacturer's recommendations and infected at low moi (0.1 or 0.01 pfu/cell) with the corresponding ECTVs. Two days after transfection/infection, the intermediate single-crossover recombinant viruses in which the complete plasmid has been inserted into the ECTV genome were selected by three to five consecutive infection rounds in the presence of 10 µg/ml puromycin and monitored by EGFP expression. Puromycin was added at 4 hpi and maintained for a 48 h period, after which virus was harvested and used as inoculum for the following selection round. Recombinant viruses (ECTVΔCD30, ECTVRevCD30 and ECTVmCD30) were finally selected by successive plaque purification of white plaques in the absence of puromycin and screening by a *CD30*-specific PCR to identify the desired recombinant viruses. The genomic structure of all recombinant viruses obtained was analyzed by PCR amplification of the *vCD30* and flanking genes, and by Southern blot to confirm absence of large genome rearrangements that may not have been noticed by PCR (not shown).

### Expression and purification of recombinant ECTV vCD30 protein

The vCD30 gene without the sequence coding for its predicted amino terminal signal peptide was amplified using oligonuclotides CD30-17 (5′-CGCGCTAGCACGTGTCCTAATGATTACTATCTTG) and CD30-18 (5′-GCGCTCGAGTCATGATGAGTATTTATGATAACAAAG) and ECTV DNA as a template. The PCR product was cloned into NheI- and XhoI-digested pRSETA (Invitrogen) to generate plasmid pAH14. The vCD30 was expressed as an N-terminal 6×His tag fusion protein in BL21 DE3 *E.coli* using plasmid pAH14H and purified by Ni-NTA (Qiagen) affinity chromatography under denaturing conditions following the manufacturer's guidelines. The purified protein was used to generate polyclonal rabbit anti-vCD30 sera. To express a C-terminally 6×His-tagged vCD30 in a eukaryotic vector, the complete vCD30 ECTV gene was amplified using oligos CD30-3 (5′-CGCAAGCTTGGATCCATGAAGATGAATACTATCTTTTTATC) and CD30-4 (5′-CGCGCGGCCGCTGATGAGTATTTATGATAACAAAG) and cloned into HindIII/NotI-digested pBac1(Novagen), generating plasmid pMS2. This was used to generate the recombinant baculovirus AcMS2 using the Novagen BacPAK system following the manufacturer's instructions.

### Biomolecular interaction analysis by SPR

Cytokine binding specificity was determined using a BIAcore X biosensor (Biacore, Uppsala, Sweden). For the ligand screening experiments, purified recombinant vCD30 protein was amine-coupled to CM5 chips to a level of aprox. 5000 RU (5000 pg/mm^2^). Recombinant murine TNFSFs were then injected at a 100 nM or 200 nM concentration in HBS-EP buffer (10 mM Hepes, 150 mM NaCl, 3 mM EDTA, 0.005% (v/v) surfactant P20; pH 7.4) at a flow rate of 10 µl/min and association and dissociation monitored. The surface was regenerated after each injection using 10 mM glycine-HCl pH 1.5. All Biacore sensorgrams were analysed using BIAevaluation 3.2 software. Bulk refractive index changes were removed by subtracting the reference flow cell responses and the average response of a blank injection was subtracted from all analyte sensorgrams to remove systematic artifacts.

### Infection of mice

Female 6–8 weeks old BALB/c OlaHsd or C57BL/6 mice (Harlan) were anesthesized using isofluorane and infected subcutaneously in the left hind footpad or intranasally with 10 µl of semipurified virus. Mice were housed in ventilated racks (Tecniplast) under biological safety level 3 containment facilities. Monitoring of infected animals was performed daily. Animals were weighed, scored for clinical signs of illness (scores ranging from 0 for healthy animals to 4 for severely diseased animals) and footpad swelling measured where appropriate. These experiments have been approved by the Biological Safety Committee of the Centro de Investigación en Sanidad Animal (CISA, INIA, Valdeolmos, Madrid) and animals are housed and handled according to local and EU legal requirements.

### Immunohistochemistry analyses

Lung and spleen samples from infected mice were removed aseptically, fixed in 10% buffered formalin solution and embedded in paraffin wax. For structural and immunohistochemical analysis, sections (3 µm) were cut and stained with H&E or processed for immunohistochemical techniques. To detect virus or ECTV vCD30 protein, formalin fixed serial sections were incubated with polyclonal rabbit anti-VACV antibody from a VACV-infected rabbit [59] or the polyclonal rabbit anti-CD30 antibody obtained in our laboratory, respectively. Secondary goat anti-rabbit immunoglobulin G (Dako) was detected using an avidin-peroxidase-complex kit (PIERCE, Thermo Scientific) and 3,3′-diaminobenzidine tetrahydrochloride (Sigma) following the manufacturer's instructions. The slides where counterstained with Mayer's haematoxylin, dehydrated, and mounted with DPX mountant (Surgipath). Specific primary antibodies were replaced by phosphate buffered saline or normal goat serum in negative control sections.
